# No evidence for adaptation to local rhizobial mutualists in the legume *Medicago lupulina*

**DOI:** 10.1002/ece3.3012

**Published:** 2017-05-10

**Authors:** Tia L. Harrison, Corlett W. Wood, Isabela L. Borges, John R. Stinchcombe

**Affiliations:** ^1^ Department of Ecology and Evolutionary BiologyUniversity of TorontoTorontoONCanada; ^2^ Centre for Genome Evolution and FunctionUniversity of TorontoTorontoONCanada

**Keywords:** coevolution, genome scan, legume, mutualism, reciprocal inoculation, rhizobia

## Abstract

Local adaptation is a common but not ubiquitous feature of species interactions, and understanding the circumstances under which it evolves illuminates the factors that influence adaptive population divergence. Antagonistic species interactions dominate the local adaptation literature relative to mutualistic ones, preventing an overall assessment of adaptation within interspecific interactions. Here, we tested whether the legume *Medicago lupulina* is adapted to the locally abundant species of mutualistic nitrogen‐fixing rhizobial bacteria that vary in frequency across its eastern North American range. We reciprocally inoculated northern and southern *M. lupulina* genotypes with the northern (*Ensifer medicae*) or southern bacterium (*E. meliloti*) in a greenhouse experiment. Despite producing different numbers of root nodules (the structures in which the plants house the bacteria), neither northern nor southern plants produced more seeds, flowered earlier, or were more likely to flower when inoculated with their local rhizobia. We then used a pre‐existing dataset to perform a genome scan for loci that showed elevated differentiation between field‐collected plants that hosted different bacteria. None of the loci we identified belonged to the well‐characterized suite of legume–rhizobia symbiosis genes, suggesting that the rhizobia do not drive genetic divergence between *M. lupulina* populations. Our results demonstrate that symbiont local adaptation has not evolved in this mutualism despite large‐scale geographic variation in the identity of the interacting species.

## 1 Introduction

Characterizing the circumstances under which local adaptation evolves informs our understanding of the relative importance of gene flow and selection, and thereby the extent and limitations of adaptive evolution (Antonovics, 1976; Bridle & Vines, 2007; Hereford, 2009; Savolainen, Lascoux, & Merilä, 2013; Whitlock, 2015). However, existing tests of local adaptation to the biotic environment focus disproportionately on antagonistic interactions (but see Anderson et al. 2004, Hoeksema & Thompson, 2007; Barrett, Broadhurst, & Thrall, 2012), limiting our understanding of adaptation within the broad suite of interspecific interactions that occur in nature. Here, we performed a reciprocal inoculation experiment to test for local adaptation in a classic mutualism: the symbiosis between legumes and nitrogen‐fixing bacteria.

Local adaptation—when native genotypes outperform foreign genotypes in their home environment (Hereford, 2009)—is driven by differences in selection in alternative environments and is reflected in divergent phenotypes and genotypes between populations. The literature on local adaptation between interacting species is dominated by antagonistic species interactions such as those between hosts and their parasites, pathogens, or prey (Brodie, Ridenhour, & Brodie, 2002; Hoeksema & Forde, 2008; Kawecki & Ebert, 2004; Koskella, Lin, Buckling, & Thompson, 2012). Direct tests of symbiont local adaptation in mutualisms are rare (Brockhurst & Koskella, 2013; Hoeksema & Forde, 2008). Nevertheless, several lines of evidence suggest that adaptation to the local mutualist is a common feature of positive species interactions. Phenotype matching between local plant and pollinator communities is pervasive (Anderson, Johnson, & Anderson, 2009; Gómez, Abdelaziz, Camacho, Muñoz‐Pajares, & Perfectti, 2009; Koski & Ashman, 2015), and a recent reciprocal translocation experiment showed that a plant's reproductive success is highest in its local pollinator community (Newman, Manning, & Anderson, 2015). In the classic mutualism between leguminous plants and nitrogen‐fixing bacteria, genotype‐by‐genotype interactions—when fitness depends jointly on the genotypes of both partners—account for a substantial proportion of genetic variation in fitness‐related traits within plant populations (Ehinger et al., 2014; Heath, 2010; Heath, Burke, & Stinchcombe, 2012). On a broad geographic scale, these interactions are predicted to manifest as symbiont local adaptation when coupled with population differences in symbiont genotype frequencies (Heath & Nuismer, 2014).

Ultimately, directly testing for symbiont local adaptation in mutualisms requires assaying the fitness consequences of sympatric and allopatric symbionts in a reciprocal inoculation experiment (Heath & Stinchcombe, 2014). The diagnostic signature of symbiont local adaptation in these experiments is a genotype‐by‐genotype interaction for fitness, indicating that the fitness of one partner depends on the identity of its symbiont (Clausen & Hiesey, 1958; Clausen, Keck, & Hiesey, 1940; Kawecki & Ebert, 2004). Although these experiments are frequently used to test for parasite local adaptation in antagonistic interactions (reviewed in Hoeksema & Forde, 2008), they are less commonly used to test for mutualist local adaptation [but see (Barrett et al., 2012; Hoeksema & Thompson, 2007; Johnson, Wilson, Bowker, Wilson, & Miller, 2010; Newman et al., 2015)].

The economically and ecologically important mutualism between legumes in the genus *Medicago* and nitrogen‐fixing bacteria (“rhizobia”) is well suited to testing for adaptation to the local mutualist (Cook, 1999; Cook, VandenBosch, de Bruijn, & Huguet, 1997; Young et al., 2011). In the facultative *Medicago*‐rhizobia symbiosis, soil bacteria in the genus *Ensifer* (formerly *Sinorhizobium*) (Young, 2010) fix atmospheric nitrogen for their plant hosts in exchange for carbohydrates and housing in specialized root organs called nodules (Mylona, Pawlowski, & Bisseling, 1995; van Rhijn & Vanderleyden, 1995). In eastern North America the relative frequencies of two principal symbionts (*Ensifer medicae* and *E. meliloti*) (Béna, Lyet, Huguet, & Olivieri, 2005) vary along a latitudinal cline (Figure S1) (Harrison, Wood, Heath, & Stinchcombe,in press [Link]), which may generate strong selection on *Medicago* populations to adapt to their local *Ensifer* species. The bacteria are essential for plant growth in nitrogen‐poor edaphic environments (Simonsen & Stinchcombe, 2014a), and genes mediating the association experience strong selection in both *Medicago* and *Ensifer* (Bailly, Olivieri, De Mita, Cleyet‐Marel, & Béna, 2006; Bonhomme et al., 2015; De Mita, Santoni, Ronfort, & Bataillon, 2007; Epstein et al., 2012). Finally, there is substantial evidence for genotype‐by‐genotype interactions for fitness traits between *Medicago truncatula* and its *Ensifer* symbionts (Gorton, Heath, Pilet‐Nayel, Baranger, & Stinchcombe, 2012; Heath, 2010; Heath et al., 2012), and suggestive evidence for some degree of co‐speciation in the two genera (Béna et al., 2005).

In this study, we performed a reciprocal inoculation experiment to test for adaptation to the local rhizobia species in the black medic (*Medicago lupulina*). We tested the effect of sympatric and allopatric rhizobia on plant fitness in a greenhouse experiment. Second, we took advantage of an existing genomic dataset and performed a genome scan for loci that exhibited elevated differentiation between field‐collected plants associated with different bacterial species in natural populations. Genome scans identify loci that exhibit heightened differentiation between populations inhabiting alternative environments, which are presumed to constitute the genetic basis of local adaptation (Coop, Witonsky, Di Rienzo, & Pritchard, 2010; Günther & Coop, 2013; Savolainen et al., 2013; Tiffin & Ross‐Ibarra, 2014). Unlike reciprocal inoculation experiments, these tests integrate across generations and ancillary environmental variation, capturing the cumulative effects of long‐term selection in alternative environments (Jensen, Foll, & Bernatchez, 2016; Tiffin & Ross‐Ibarra, 2014; de Villemereuil, Gaggiotti, Mouterde, & Till‐Bottraud, 2015).

However, neither the phenotypic nor genomic approaches revealed strong evidence of adaptation to the local rhizobia in *M. lupulina*, suggesting that symbiont local adaptation has not evolved in this mutualism's North American range.

## 2 Materials and Methods

### 2.1 Study system

*Medicago lupulina* is an annual, highly self‐fertilizing legume native to Eurasia (Turkington & Cavers, 1979; Yan, Chu, Wang, Li, & Sang, 2009). After its introduction to North America in the 1700s, *M. lupulina* expanded its range to occupy nitrogen‐poor areas of the continent's temperate and subtropical regions (Turkington & Cavers, 1979). In eastern North America, the relative frequencies of *M. lupulina*'s two symbiotic rhizobia species (*Ensifer medicae* and *E. meliloti*) vary along a northwest‐to‐southeast cline (Figure S1) (Harrison, Wood, Heath, & Stinchcombe, in press[Link]). *Medicago* has a short generation time (Turkington & Cavers, 1979), its rhizobia are easily manipulated (Heath & Tiffin, 2007), an annotated genome is available in the genus (Young et al., 2011), and the genes involved in the rhizobial mutualism are extensively characterized (Cook et al., 1997; Mylona et al., 1995; Young et al., 2011).

### 2.2 Reciprocal inoculation experiment

To test for adaptation to the local rhizobia, we inoculated *M. lupulina* genotypes from the northern and southern portions of the plant's eastern North American range with either the locally abundant rhizobium species in the north (*E. medicae*) or in the south (*E. meliloti*). From a total of 39 *M. lupulina* populations sampled between Delaware and Ontario in September‐October of 2013 (Harrison, Wood, Heath, & Stinchcombe,in press [Link]), we selected seven southern and seven northern plant populations in which Harrison (in press[Link]) detected only a single *Ensifer* species (Figure 2, Table S1; see Figure S1 for a complete map with all 39 sampled populations). Within each population, seeds and root nodules were collected from 2 to 10 randomly chosen *M. lupulina* individuals. All sampled plants were at least 0.5 m apart. Nodules were stored at 4°C in plastic bags until they were processed. Field‐collected seeds from these populations were grown in the greenhouse for one generation to reduce maternal and environmental effects from the field, and we performed our experiments using the progeny of these greenhouse‐grown plants.

**Figure 1:**
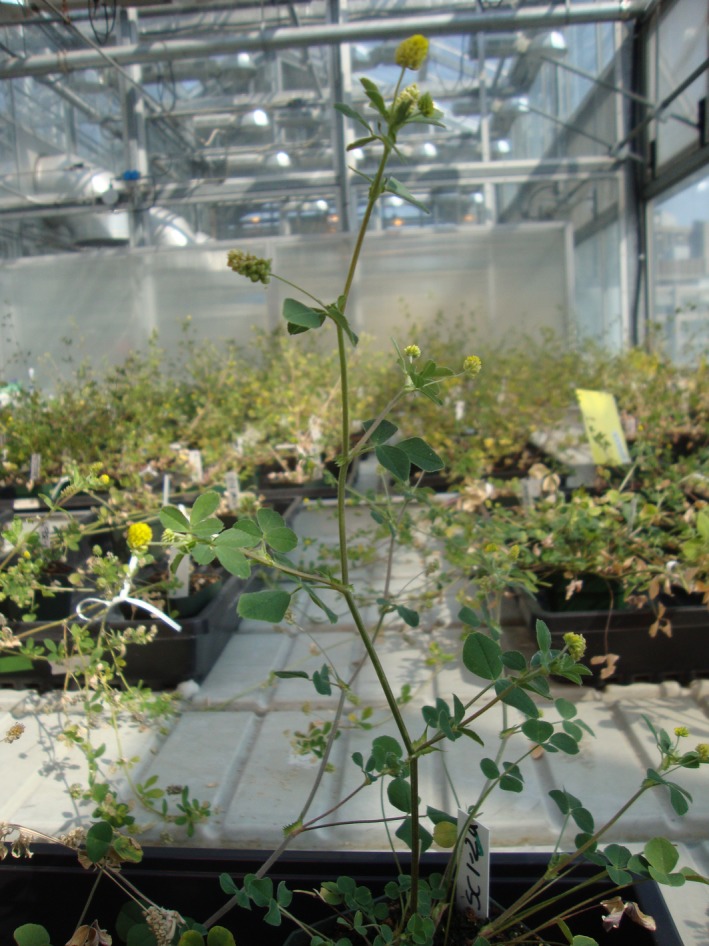
A *Medicago lupulina* individual flowering in the greenhouse

We planted F_1_ greenhouse‐derived seeds of 43 maternal families (27 from the north and 16 from the south) in a split‐plot randomized complete block design in the greenhouse at the University of Toronto. Each block was divided into two bacterial treatments, each containing 15 northern and 11 southern plants, the locations of which were randomized within blocks. Populations were split across blocks. Due to seed limitations, not all families were represented in every block, but within a block both bacterial treatments comprised the same 26 families. We replicated this design across six blocks, for a total of 312 plants (6–13 replicates per family for 37 families; 1–4 replicates per family for six families). An additional block containing 42 plants (33 from the north and nine from the south) served as an inoculation control, and a means for estimating plant performance and fitness in the absence of either bacterial species. Prior to planting, seeds were scarified with a razor blade, sterilized with ethanol and bleach, and stratified on 8% water agar plates at 4°C for 7 days to germinate. We planted with sterile forceps into cone‐tainers filled with sand (autoclaved twice at 121°C). We misted seedlings with water daily and fertilized with 5 ml of nitrogen‐free Fahraeus medium (noble.org/medicagohandbook) twice before inoculation with rhizobia.

The *Ensifer* strains used for inoculation were recovered from frozen samples collected by Harrison et al.( in press[Link]) from two of the populations used in our experiment. The strains were originally cultured from field‐collected root nodules by sterilizing one nodule per plant in ethanol and bleach, and crushing and plating it onto a 2% tryptone yeast (TY) agar plate. Strains were re‐streaked onto TY agar four times to reduce contamination and grown at 30°C for 48 hr, after which they were transferred to liquid TY media and cultured for 2 days at 30°C. To identify each strain to species (*E. medicae* or *E. meliloti*), DNA was extracted from liquid cultures (cell density: 8 × 10^8^ cells/ml) using the MoBio UltraClean Microbial DNA Isolation Kit, whole‐genome sequenced at SickKids Hospital (Toronto, Ontario), and genotyped using GATK (McKenna et al., 2010). We used alignment scores and the *Ensifer* 16S locus (Rome, Cleyet‐Marel, Materon, Normand, & Brunel, 1997) to determine species identity of rhizobia strains associated with the sampled plants.

**Figure 2:**
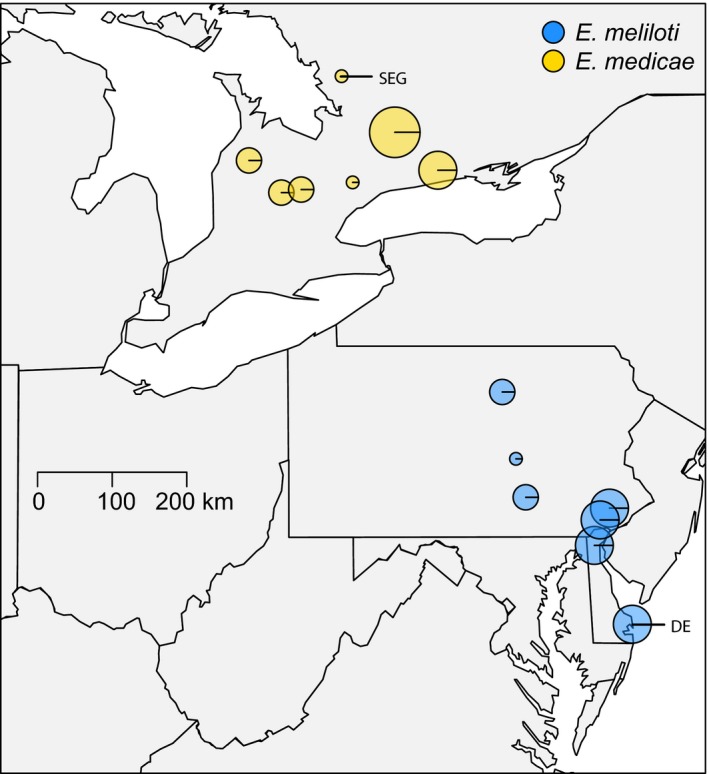
Locations of the 14 *Medicago lupulina* populations used in this study. The size of each circle corresponds to the number of plants sampled from the population, and the color indicates the rhizobia. The *Ensifer medicae* strain used in the reciprocal inoculation experiment was obtained from the northernmost population sampled (“SEG”); the *E. meliloti* strain was obtained from the southernmost population (“DE”). See Table S1 for GPS coordinates

We selected one *E. medicae* strain from the northernmost population in Ontario and one *E. meliloti* strain from the southernmost population in Delaware for our experiment (“SEG” and “DE” in Figure 2). Genetic diversity is very low among strains within *Ensifer* species across North America (Harrison, Wood, Heath, & Stinchcombe,in press [Link]), so the specific strains used are not likely to influence our results. Prior to inoculation, these strains were cultured as described above from samples stored at −80°C. Liquid cultures were diluted with sterile TY media to an OD600 reading of 0.1 (a concentration of ~10^6^ cells/ml) (Simonsen & Stinchcombe, 2014b). Each plant received 1 ml of inoculate 13 days after planting and 1 ml again 10 days later. Controls were also inoculated twice with sterile TY media 10 days apart and were used to assess rhizobia contamination across treatments. Throughout the remainder of the experiment, all plants were bottom‐watered three times a week. We used two bottom‐watering trays per block, such that all plants in a given bacterial treatment had the same tray, while those from the alternative bacterial treatment had a different tray.

We scored mortality weekly throughout the experiment, counted the number of leaves every 4 weeks, recorded the date of first flower, and collected seeds. After 5 months, which approximates the length of the April‐October growing season in southern Ontario (Turkington & Cavers, 1979), we harvested all plants and collected any remaining unripe seeds. We dried and weighed aboveground tissue from each plant to the nearest 0.1 mg and counted all seeds and root nodules (symbiotic organs housing the rhizobia).

We analyzed five traits to test whether northern and southern *M. lupulina* plants were adapted to their local rhizobium: number of seeds, aboveground biomass, flowering time (excluding plants that did not flower), probability of flowering, and number of nodules. All analyzes were performed in R v.3.2.4 with sum‐to‐zero contrasts (“contr.sum”) (R Core Team, 2016), and we tested significance using type III sums of squares in the function ANOVA in the *car* package (Fox & Weisberg, 2011). Log‐transformed aboveground biomass and flowering time were analyzed with general linear mixed models using the function lmer in the *lme4* package (Bates, Mächler, Bolker, & Walker, 2015). Probability of flowering and number of nodules were analyzed with generalized linear mixed models with binomial and Poisson error distributions, respectively, using the function glmer in the *lme4* package (Bates et al., 2015). We verified that the residuals from all models met the assumptions of linearity, normality, and homoscedasticity through visual inspection of quantile–quantile plots, plots of the residuals versus fitted values, and scale‐location plots. Seed number was severely zero‐inflated (42% of plants did not produce seeds), so we analyzed it using a mixture model.

Each of the above models included rhizobia treatment (*E. medicae* or *E. meliloti*), region (north or south), and the rhizobia‐by‐region interaction as fixed effects. A significant rhizobia‐by‐region interaction, in which northern plants have higher fitness when inoculated with *E. medicae* and southern plants have higher fitness with *E. meliloti*, would be evidence for symbiont local adaptation. We included a fixed effect of researcher in our analysis of nodule counts. Block, population, and family nested within population were included as random effects. We also included the block‐by‐treatment interaction as a random effect because the rhizobia treatment was applied at the half‐block rather than at the plant level (Altman & Krzywinski, 2015). While this design provides a weaker test of the rhizobia main effect, it is sensitive to the detection of rhizobia‐by‐region interactions, the main goal of our experiment (Altman & Krzywinski, 2015).

We analyzed seed number with a zero‐inflated Poisson model implemented with the function MCMCglmm in the package *MCMCglmm* (Hadfield, 2010). Zero‐inflated models are a type of mixture model in which the zero class is modeled as the combined result of binomial and count processes (Zuur, Ieno, Walker, Saveliev, & Smith, 2009). In MCMCglmm, zero‐inflated Poisson GLMMs are fit as multiresponse models with one latent variable for the binomial zero‐generating process and one for the Poisson count‐generating process (Hadfield, 2015). We fit a model for seed number that included fixed effects of rhizobia, region, the rhizobia‐by‐region interaction, and the reserved MCMCglmm variable “trait” that indexes the binomial and Poisson latent variables. We omitted the interaction between trait and other fixed effects to estimate a single effect of rhizobia, region, and the rhizobia‐by‐region interaction across both the binomial and Poisson processes. Block, population, family, and the block‐by‐treatment effect were included as random effects. Different random effect variances were fit to the binomial and Poisson processes using the “idh” variance structure in MCMCglmm (Hadfield, 2015). We fit a residual variance (R) structure using the argument rcov = ~us(trait):units, which allows a unique residual for all predictors in the model, used the default priors for the fixed effects (mean = 0, variance = 10^10^) and specified parameter‐expanded priors (alpha.mu = 0, alpha.v = 1,000) for the random effects (Hadfield, 2010).

We ran the model for 1,300,000 iterations, discarded the first 300,000 iterations, and stored every 1,000th iterate. Model convergence was assessed with traceplots, running mean plots, and autocorrelation plots of the fixed and random effects using the *coda* (Plummer, Best, Cowles, & Vinces, 2006) and *mcmcplots* (McKay Curtis, 2015) packages. Even though we used parameter‐expanded priors on the random effects, the estimates of the population and block random effects remained close to zero, but omitting these terms from our model did not qualitatively change the results.

Finally, we calculated pairwise correlations between all traits using Spearman's correlation on the family means for each trait. We obtained family means for biomass, flowering time, and number of nodules by extracting the conditional modes (also known as the best linear unbiased predictors, or BLUPs) for each level of the family random effect from the models described above. For number of seeds, we used the marginal posterior modes of the family random effect as our family mean estimates.

### 2.3 Genomic dataset

A limitation of using reciprocal inoculation experiments to test for symbiont local adaptation is that the fitness benefit of a symbiosis often depends on the biotic and abiotic environmental conditions in which it is expressed (Barrett et al., 2012; Heath, Stock, & Stinchcombe, 2010; Heath & Tiffin, 2007; Porter, Stanton, & Rice, 2011; Simonsen & Stinchcombe, 2014a). To address this limitation, we took advantage of a pre‐existing *M. lupulina* SNP dataset collected by Harrison et al. (in press[Link]) to perform genomic scans in *M. lupulina*. The goal of this analysis was to determine whether genes involved in the legume–rhizobia symbiosis are differentiated between plants associated with different *Ensifer* species in natural populations, a pattern that would be consistent with symbiont local adaptation.

Details on SNP discovery methods can be found in Supplemental Methods (Appendix S1), and Harrison et al. (in press[Link]). In brief, field‐collected seeds from 73 *M. lupulina* individuals were grown in the greenhouse as described in the “Reciprocal inoculation experiment” section above. We extracted DNA from leaf tissue collected from one individual per maternal line and samples were sequenced at Cornell University using genotyping‐by‐sequencing (GBS) in two Illumina flow cell lanes (Elshire et al., 2011). Genomic libraries were prepared with the restriction enzyme EcoT22I, and SNPs were called using the program Stacks (Catchen, Hohenlohe, Bassham, Amores, & Cresko, 2013; Catchen et al., 2011). We extracted and sequenced rhizobia DNA from one nodule from each field‐sampled plant and determined the species identity of each strain as described in the “Reciprocal inoculation experiment” section above.

We searched for outlier loci between *M. lupulina* plants hosting *E. medicae* and *E. meliloti* to assess whether there is evidence for genetic divergence between plants associated with different *Ensifer* species. We used the program Bayenv2 to calculate X^T^X statistics for each SNP in the *M. lupulina* sample (Coop et al., 2010): X^T^X is an F_ST_‐like statistic that controls for population variation and covariation in allele frequencies (i.e., population structure). We estimated the covariance matrix using 100,000 iterations. Because we only wanted to calculate X^T^X statistics and did not wish to calculate environmental correlations, we included an environmental file of dummy values to run Bayenv2 but avoid environmental analysis. We ranked SNPs from highest to lowest X^T^X values and identified the top 1% of SNPs to BLAST against the reference genome of *M. truncatula* to identify the outlier loci involved in rhizobia association in *M. truncatula* (taxonomy ID 3880) (Tang et al., 2014). We used nucleotide BLAST (blastn) to search somewhat similar sequences in the unannotated *M. truncatula* genome in order to retrieve chromosome positions for our outlier loci. To identify the orthologous gene associated with each outlier locus, we then looked up the chromosome position of each outlier in the annotated *Medicago truncatula* genome (Mt. 4.0 http://jcvi.org/medicago/). We performed the outlier loci test in three ways. First, we characterized outlier loci using the range‐wide sample of plants (73 plant individuals) and compared the results to outlier loci found using the southern Ontario samples (49 plant individuals) to account for possible covariance between environmental gradients and bacterial species composition. Second, because outlier loci are usually in linkage disequilibrium (LD) with the causal genes responsible for adaptation, we searched for legume–rhizobia symbiosis genes within 5 and 10 kb of our detected outlier loci (Branca et al., 2011). Third, we measured the distance in base pairs between the *M. truncatula* orthologs of detected outlier loci and key *M. truncatula* genes involved in the rhizobia symbiosis, assuming synteny between *M. lupulina* and *M. truncatula*. Details of these analyses can be found in the Supplemental Methods (Appendix S2).

## 3 Results

### 3.1 Reciprocal inoculation experiment

Uninoculated *Medicago lupulina* plants performed extremely poorly without rhizobia. None of our uninoculated control plants flowered or set seed, and the biomass of control plants was approximately 20‐fold smaller than inoculated plants (least squares mean ± SE (mg): controls: 21.01 ± 0.05; inoculated plants from both rhizobia treatments: 476.01 ± 0.03; *F*_1,14.808_ = 610.7, *p* < .001). The performance of the control plants also demonstrates that cross‐contamination between the two rhizobia treatments was likely minimal in our experiment. Only one of 42 uninoculated control plants produced nodules, and this anomalous individual was similar in size to the rest of the controls for the first several months, indicating that it probably did not nodulate until late in the experiment.

In plants inoculated with *E. medicae* or *E. meliloti*, pairwise family mean correlations between all measured traits were generally low, indicating that the traits that we measured were largely independent of one another (*r* ≤ |.10|, *p* ≥ .54). Only flowering time and aboveground biomass were significantly correlated (*r* = .49, *p* = .002); later‐flowering plants had greater aboveground biomass.

**Figure 3:**
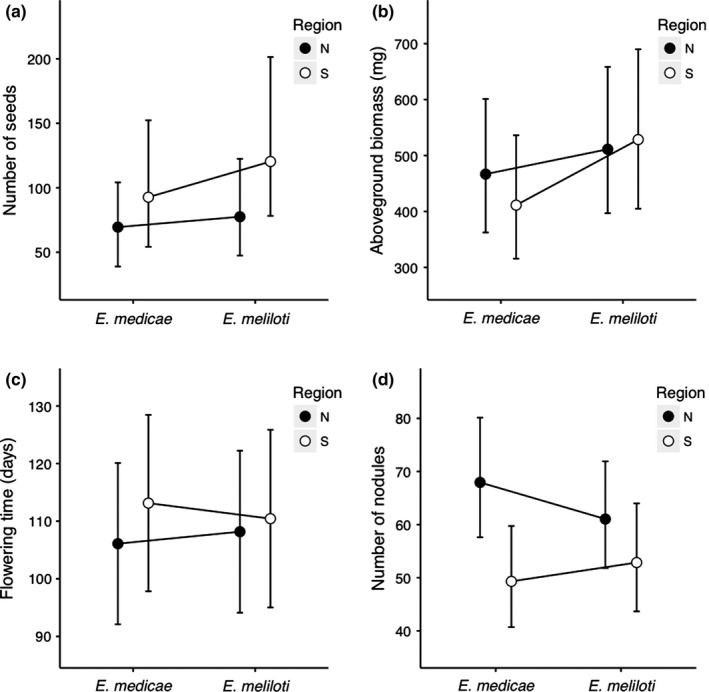
Least squares means and 95% confidence intervals for northern (black) and southern (white) plants grown in the two rhizobia treatments. *Ensifer medicae* is the locally abundant rhizobia in the north, and *E. meliloti* is the locally abundant rhizobia in the south. (a) Number of seeds; (b) aboveground biomass; (c) flowering time; (d) number of nodules

Our analysis of seed number, probability of flowering, and flowering time revealed no evidence of adaptation to the local rhizobia. There was no significant rhizobia‐by‐region interaction for any of these reproductive traits (Figure 3, Table 1). There was a marginally significant effect of region on seed number; southern plants produced more seeds than northern plants in both rhizobia treatments (Figure 3, Table 1). There was no significant effect of rhizobia treatment or region on either flowering trait (Figure 3, Table 1).

**Table 1:** Results of general(ized) linear mixed models testing for local adaptation in the reciprocal inoculation experiment

				pMCMC
Seeds (MCMC GLMM)	Rhizobia			0.204
	Region			0.070
	Rhizobia × region			0.350

		*F*	*df*	*p*
Biomass (LMM)	Rhizobia	1.955	1, 5.097	0.220
	Region	0.131	1, 12.782	0.723
	Rhizobia × region	3.747	1, 248.656	0.054
Flowering time (LMM)	Rhizobia	0.016	1, 5.436	0.903
	Region	0.252	1, 12.896	0.624
	Rhizobia × region	1.378	1, 164.795	0.242

		Wald χ^2^	*df*	*p*
Prob. of flowering (GLMM)	Rhizobia	0.012	1	0.912
	Region	0.047	1	0.829
	Rhizobia × region	0.231	1	0.631
Nodules (GLMM)	Rhizobia	0.107	1	0.743
	Region	5.581	1	0.018
	Researcher	95.079	1	<0.001
	Rhizobia × region	34.806	1	<0.001

The type of model used is indicated below each trait. GLMM: generalized linear mixed model (see text for error distribution). LMM: Linear mixed model (Gaussian error).

The rhizobia‐by‐region interaction for aboveground biomass was marginally significant (P_rhizobia‐by‐region interaction_ = 0.054, Table 1). While the biomass of northern plants was unaffected by rhizobia treatment, southern plants produced more aboveground biomass when inoculated with *E. meliloti* (Figure 3), the locally abundant rhizobia in south.

We found a highly significant rhizobia‐by‐region interaction for nodule number (Table 1). Northern plants produced more nodules than southern plants when inoculated with *E. medicae*, the locally abundant rhizobia in the north. The difference between northern and southern plants decreased when inoculated with *E. meliloti*, an effect that was driven by both an increase in nodulation in southern plants and a decrease in nodulation in northern plants (Figure 3). There was also a significant effect of region, indicating that northern plants produced more nodules across both rhizobia treatments, and a significant effect of researcher (Table 1).

### 3.2 Genomic outlier analysis

We identified three outlier loci that appeared in the top 1% of SNPs in both the range‐wide *M. lupulina* sample and southern *M. lupulina* Ontario sample in our Bayenv2 analysis (Supplemental Table S2 and S3). None of these three loci mapped to a specific gene in the *M. truncatula* reference genome. Furthermore, we did not find any genes involved in the legume–rhizobia interaction within 5 or 10 kb of our three outlier loci (Table S4). Finally, the base pair distances between our outlier loci and known genes involved in the *Medicago‐Ensifer* mutualism were very large (minimum: 18 kb) (Table S4). Details on summary X^T^X statistics, BLAST alignment scores, and gene functions are presented in the Supplemental Methods (Appendix S3) and Table S2.

## 4 Discussion

We performed a reciprocal inoculation experiment to test for symbiont local adaptation of *M. lupulina* to its mutualistic nitrogen‐fixing bacteria across its eastern North American range. We found no evidence for adaptation to the locally abundant rhizobia species for the majority of traits, including our best proxy for fitness (number of seeds). Our analysis of pre‐existing genomic data produced similar results. None of the well‐characterized legume–rhizobia symbiosis genes were differentiated between field‐collected plants associated with different rhizobia. Our results suggest that local rhizobia do not have differential fitness consequences for their host plants, nor do they drive genetic divergence in known symbiosis genes. Symbiont local adaptation is either absent or weak in this mutualism's eastern North American range despite the strong cline in the relative abundances of the two rhizobia species.

### 4.1 Reciprocal inoculation experiment and genomic outlier analysis

Uninoculated plants performed extremely poorly without either *Ensifer* species, demonstrating that *M. lupulina* is adapted to symbiosis with rhizobia. Despite differential nodulation with local and foreign rhizobia (P_rhizobia‐by‐region_ < 0.001, Table 1), however, there was no strong evidence for adaptation to the local rhizobia in other plant traits. One explanation for this pattern is that plants modify their nodulation strategy to compensate for differences in symbiotic efficiency with local and foreign rhizobia. The congeneric species *M. truncatula* adjusts its nodulation strategy in response to the rhizobia nitrogen fixation efficiency (Heath & Tiffin 2009), which jointly depends on plant and rhizobia genotype (Mhadhbi, Jebara, Limam, Huguet, & Aouani, 2005). If plants produce more nodules with less efficient symbionts, increased nodulation may not translate to greater nitrogen uptake, masking any effects of differential nodulation on biomass and seed production. The fact that seed number, a reasonable proxy for total fitness in a selfing annual or short‐lived perennial like *M. lupulina* (Turkington & Cavers, 1979), was unaffected by the local rhizobia strongly suggests that adaptation to the local rhizobia was absent in our experiment at the whole‐plant level.

Even in the traits that exhibited a rhizobia‐by‐region interaction—the statistical signature of local adaptation—the data are only weakly consistent with the canonical pattern of local adaptation. The strongest test of local adaptation is whether local genotypes outperform foreign genotypes in all environments (the “local‐versus‐foreign” criterion) (Kawecki & Ebert, 2004). Neither trait that exhibited any rhizobia‐by‐region interaction (number of nodules and aboveground biomass) satisfied this criterion. Instead, our results were more closely aligned with a weaker test of local adaptation, which diagnoses local adaptation when each genotype's fitness is greater in its native environment than in alternative environments (the “home‐versus‐away” criterion) (Kawecki & Ebert, 2004).

Although reciprocal inoculation experiments are powerful because they reflect whole‐organism performance in native and foreign environments, genotype‐by‐environment interactions are sensitive to experimental conditions (Kawecki & Ebert, 2004) and null results from any single experiment could be due to experimental conditions not adequately reflecting the typical natural environment (in our case, cone‐tainers, sterilized greenhouse soil, artificial day length control, absence of other biotic interactors, etc.). Because our reciprocal inoculation experiment produced no evidence for symbiont local adaptation in *M. lupulina*, we took advantage of a pre‐existing genomic dataset to perform a genomic outlier analysis. Our genome scan should circumvent the weaknesses inherent in reciprocal inoculation experiments, because it detects allele frequency differences between plants hosting different rhizobia integrated across many generations of selection and ancillary environmental variation.

We also found very weak evidence of symbiont local adaptation in the outlier analysis. The loci that were highly differentiated between plants hosting different *Ensifer* species (the top 1% of loci in the X^T^X outlier analysis) were not associated with any genes involved in the legume–rhizobia symbiosis in either the range‐wide or Ontario samples. Moreover, none of the *M. truncatula* orthologs of our outlier loci were located within the scale of linkage disequilibrium (5–10 kb in *M. truncatula*) (Branca et al., 2011) from known symbiosis genes. It is unlikely that the loci identified in our genome scan are novel *M. lupulina*‐specific symbiosis genes underlying adaptation to the local bacteria. The *Medicago* genes involved in symbiotic interactions with rhizobia are well‐characterized and highly conserved in legumes (Branca et al., 2011; De Mita, Santoni, Hochu, Ronfort, & Bataillon, 2006; Gorton et al., 2012; van Rhijn & Vanderleyden, 1995; Rostas, Kondorosi, Horvath, Simoncsits, & Kondorosi, 1986; Stanton‐Geddes et al., 2013). *Medicago lupulina* is a close relative of *M. truncatula* (Bena, 2001; Yoder et al., 2013), and both plants fix nitrogen with both *Ensifer* species tested in our experiment (Béna et al., 2005). However, our results are subject to the caveats of genome scans for selection (Pavlidis, Jensen, Stephan, & Stamatakis, 2012). In particular, our sample size in terms of individuals, and the number of SNPs, was low, reducing our power. Therefore, our genome scan might not have been able to detect highly differentiated loci important for symbiont local adaptation in *M. lupulina*.

### 4.2 Local adaptation in the legume–rhizobia symbiosis

Our phenotypic and genomic data indicate that *M. lupulina* is not adapted to the local rhizobia across its eastern North American range. The absence of symbiont local adaptation in this mutualism is surprising given that the system is characterized by several features that ordinarily strongly favor its evolution. Genotype‐by‐genotype interactions commonly occur between a congener, *M. truncatula*, and different strains of the same *Ensifer* species (Heath, 2010; Heath & Tiffin, 2007; Heath et al., 2012), suggesting that the genetically divergent rhizobia *species* (Bailly et al., 2006) we assayed would have even greater effects on their plant host. Furthermore, there is a cline in the frequencies of the two rhizobia across a large geographic scale that coincides with plant population genetic structure (Harrison, Wood, Heath, & Stinchcombe, in press). What might account for the lack of symbiont local adaptation in this mutualism?

Gene flow may overwhelm the effects of local selection, leading to a low equilibrium level of genetic differentiation between plants associated with different rhizobia (McKay & Latta, 2002). Although there is a strong geographic cline in the frequencies of the two *Ensifer* species, Harrison ([Link]) did detect *E. meliloti* in some northern populations and *E. medicae* in some southern populations. Symbiont local adaptation within *M. lupulina* populations could be swamped by gene flow from neighboring populations that encounter the alternative mutualist, or by the invasion of the alternative mutualist itself. Horizontal gene transfer between the two rhizobia could similarly homogenize any signature of local selection (Bailly, Olivieri, Brunel, Cleyet‐Marel, & Béna, 2007; Lenormand, 2002). Bacteria that form nitrogen‐fixing symbioses with legumes have been shown to horizontally transfer genes involved in forming and maintaining the mutualism (Aoki, Ito, & Iwasaki, 2013; Lemaire et al., 2015; Suominen, Roos, Lortet, Paulin, & Lindström, 2001), which could largely eliminate among‐symbiont differences from the perspective of the legume host. Finally, temporal variation in the biotic and abiotic environment may modify the costs and benefits of the mutualism (Heath & McGhee, 2012; Heath et al., 2010; Simonsen & Stinchcombe, 2014a), weakening selection favoring local rhizobia.

Alternatively, symbiont local adaptation may generate relatively weak fitness trade‐offs in mutualisms. The fitness trade‐offs that are the hallmark of local adaptation evolve whenever adaptation to one environment results in maladaptation to another (Kawecki & Ebert, 2004). It has been hypothesized that selection in coevolving mutualisms strongly favors general compatibility and the reduction of fitness trade‐offs (Barrett et al., 2012; Law & Koptur, 1986; Parker, 1999). Selection to minimize fitness trade‐offs may be especially strong in the legume–rhizobia mutualism, which is crucial for plants growing in nitrogen‐poor soils (Heath et al., 2010). Under nitrogen‐limited conditions, the cost of maladaptation to a locally rare rhizobium may be severe enough to outweigh the selective advantage of a marginal increase in the benefits obtained from the locally abundant rhizobium (Barrett et al., 2012). However, this process should minimize plant–rhizobia interactions for fitness *within* rhizobia species as well, inconsistent with the pervasive genotype‐by‐genotype interactions documented between *M. truncatula* and *E. meliloti* (Heath et al., 2012).

Finally, symbiont local adaptation may be restricted to the rhizobia in this mutualism; the rhizobia may be adapted to their local *M. lupulina* genotype even though the plant does not appear to be adapted to its local rhizobium. The strongest signature of local adaptation in our reciprocal inoculation experiment occurred in nodule traits, a pattern that has also been documented in congeneric *Medicago* species (Porter et al., 2011). Differential nodulation may impact the rhizobia more than the plant, given that nodule number is correlated with rhizobia fitness in *Medicago* (Heath, 2010). Stronger symbiont local adaptation in one partner commonly occurs in host–parasite systems (Hoeksema & Forde, 2008), but the phenomenon has not been systematically explored in the context of mutualism even though asymmetrical evolutionary rates in coevolving species pairs are expected in both mutualisms and antagonisms (Bergstrom & Lachmann, 2003).

### 4.3 Complementarity of phenotypic and genotypic approaches

In the present study, we took advantage of a pre‐existing genomic dataset to complement and extend our test for symbiont local adaptation using a classic reciprocal inoculation experiment. Our genomic outlier analysis also did not produce evidence of symbiont local adaptation, possibly because of our low sample size and low SNP coverage in our dataset. However, we believe that combining an experimental approach and genomics is an innovative and powerful way to test for local adaptation that should be applied more broadly. Although genome scans and reciprocal inoculation experiments are typically treated as alternatives because they draw on fundamentally different data, together the two approaches constitute a rigorous test for local adaptation in environmentally sensitive symbioses such as the legume–rhizobia mutualism. Combined, the two approaches integrate over the effects of all loci in the genome (reciprocal inoculation experiments) and across ancillary environmental variation (genome scans), producing inferences that are less vulnerable to the weaknesses of either method (Buehler, Holderegger, Brodbeck, Schnyder, & Gugerli, 2014; Jensen et al., 2016; de Villemereuil et al., 2015). Studies of (symbiont) local adaptation should consider pairing phenotypic and genomic approaches to validate their results with independent lines of evidence and exclude alternative interpretations of the data (Jensen et al., 2016; de Villemereuil et al., 2015). Future directions for our research could include repeating the genomic outlier test with a higher quality genomic dataset to determine whether the reciprocal inoculation and genome scan produce concordant results on symbiont local adaptation.

## Conflict of Interest

None declared.

## Data Accessibility

DNA sequences for M. lupulina are uploaded to the Sequence Read Archive of NCBI (Bioproject PRJNA378787 and Biosample SAMN06562075). The VCF file for M. lupulina samples is uploaded to Dryad, doi:10.5061/dryad.77k64 (https://doi.org/10.5061/dryad.77k64). DNA sequences for the rhizobia are uploaded to the Sequence Read Archive of NCBI (Ensifer Bioproject PJRNA378842 and Biosample SAMN06562698). GPS coordinates of sampled plant and rhizobia populations are reported in Table S1.
