# Supplementary material for: No evidence for adaptation to local rhizobial mutualists in the legume Medicago lupulina
Source: Ecol Evol. 2017 May 10;7(12):4367–76. doi: 10.1002/ece3.3012 (PMC5478075; doi:10.1002/ece3.3012)
Supplement: Supplementary file 1 [file ECE3-7-4367-s001.docx]

**Supplementary Methods**

**Appendix 1**

*Bioinformatics and SNP discovery in* Ensifer

We aligned forward and reverse rhizobia reads to the reference genome of *E. meliloti* strain 1021 (Galibert *et al.*, 2001) (NCBI references chromosome AIL591688, plasmid a AE006469, plasmid b AL591985) and the *E. medicae* strain WSM419 (Reeve *et al.*, 2010) (NCBI references chromosome 150026743 plasmid b 150030273, plasmid a 150031715, accessory plasmid 150032810) using BWA (Li and Durbin, 2009) and Stampy (Lunter and Goodson, 2011) with default parameters and the bamkeepgoodreads parameter. We assigned bacterial species using a combination of the percentage of reads mapping to one reference genome, and sequences at the 16S rDNA locus (NCBI gene references 1234653 and 5324158, respectively), which differs between *E. medicae* and *E. meliloti* (Rome *et al.*, 1997). We used Integrative Genomics Viewer to visualize and check alignment quality (Robinson *et al.*, 2011). In general, 69.99 – 94.02% (median 84.71%) of reads per sample mapped to the *E. meliloti* reference genome, and 69.32 – 92.48% (median 83.49%) mapped to the *E. medicae* genome.

We used PICARD tools to format, sort, and remove duplicates in sequence alignments. We applied GATK version 3 indel realignment and GATK Unified Genotyper SNP discovery on all bacteria alignments (McKenna *et al.*, 2010) with ploidy set to haploid. We used the Select Variants parameter in GATK to select SNP variants only. We used standard hard filtering parameters and variant quality score recalibration on SNP discovery according to GATK Best Practices (DePristo *et al.*, 2011; Van der Auwera *et al.*, 2013). We filtered rhizobia SNPs for a minimum read depth (DP) of 20, a maximum DP of 226 for *E. meliloti* (230 for *E. medicae*), and a genotype quality (GP) of 30 using vcftools (Danecek *et al.*, 2011). We removed indels and sites with more than 10% of missing data from both *E. meliloti* and *E. medicae* data files. We identified synonymous SNPs using SnpEff (Cingolani, Platts, *et al.*, 2012b) and SnpSift (Cingolani, Patel, *et al.*, 2012a), using reference files GCA_000017145.1.22 and GCA_000006965.1.22 (for *E. medicae* and *E. meliloti*, respectively) in the pre-built database. We used the ANN annotation parameter in SnpSift to identify SNPs as synonymous variants and missense variants.

*Bioinformatics and SNP discovery in* Medicago

We called *Medicago* SNPs in GBS samples by following the three-stage pipeline in the program Stacks (Catchen *et al.*, 2011; 2013): cleaning raw data, building loci, and identifying SNPs. We trimmed reads to 64 bp and filtered reads by a phred score of 33, the default value for GSB reads sequenced on Illumina 2000/2500 machine. We built loci for *M. lupulina* using the *de novo* approach in Stacks (denovo_map command), setting the –m parameter at 5, the –M parameter at 1, and the -n parameter at 1. In the final stage of the pipeline, we identified SNPs under the populations command by setting the –m parameter at 5. We filtered SNPs by removing indels, removing sites with more than 10% of missing data, and removing sites that were less than 64 bps apart with vcftools (Danecek *et al.*, 2011). We also excluded 9 SNPs with heterozygosity that was higher than expected under Hardy-Weinberg.

**Appendix 2**

*Genomic outlier tests*

We first performed the BLAST test in two ways: first using the range-wide sample of plants that hosted different bacterial species (73 plant individuals), and second, focusing on southern Ontario samples (49 plant individuals). We performed the latter test because of the possibility that many loci unrelated to bacterial specificity (e.g., climatic adaptation) could be differentiated between southern Ontario and the mid-Atlantic United States due to environmental gradients that covary with bacterial species composition.

Outlier loci detected in genotyping-by-sequencing (GBS) data are rarely the actual loci responsible for adaptation; instead, they are usually in linkage disequilibrium (LD) with the causal genes. To account for this possibility, we then searched for genes involved in the legume-rhizobia symbiosis within either 5 or 10 kb of the *M. truncatula* orthologs of the outlier loci that we detected in both the range-wide and Ontario samples. This approach assumes synteny between *M. truncatula* and *M. lupulina*. We chose 5 and 10 kb based on the scale of LD in *M. truncatula* (Branca *et al.* 2011). While the scale of LD between even closely related species is likely to differ based on mutation rates, recombination, population structure, and a host of other demographic and evolutionary factors, we viewed this approach as superior to simply confining our searches to the GBS loci without accounting for their potential LD with causal genes.

Finally, we measured the distance between the *M. truncatula* orthologs of the outlier loci that we detected in both the range-wide and Ontario samples and key *M. truncatula* genes involved in the rhizobia symbiosis (again assuming synteny between *M. truncatula* and *M. lupulina*). We considered genes involved in the initial signal exchange between the legume and rhizobia (NSP, IPD3, and DMI1-DMI3); genes involved in infection thread development (LIN); and genes involved in both rhizobia signaling and infection (NFP, LYK3, and NIN) (Jones *et al.* 2007; Oldroyd *et al.* 2009; Young *et al.* 2011; Oldroyd 2013; Tang *et al.* 2014).

**Appendix 3**

*Genomic outlier results*

We identified a distribution of X^T^X statistics around the null expectation of X^T^X = 2, reflecting the 2 populations assigned in Bayenv2 (*M. lupulina* plants hosting *E. medicae* and plants hosting *E. meliloti*)*.* In the range-wide sample, 16% (354 of 2209) of SNPs had X^T^X scores greater than the null expectation of 2; in the Ontario sample, 29% (573 of 1977) of SNPs had X^T^X scores greater than 2. We detected a range of alignment scores when we used BLAST to align outlier loci with top X^T^X statistics from the whole sample and the Ontario sample to the *M. truncatula* reference genome (Table 2). The loci mapped to several different chromosomes in the *M. truncatula* reference genome.

Of the top 1% of SNPs detected in the range-wide sample (20 SNPs total), eight were associated with a specific *M. truncatula* gene (BLAST scores: 35.6 – 102; E value: 1.00e-19 – 0.31). Higher BLAST scores reflect higher-quality alignments; these scores indicate that our sequences generally aligned moderately well to the *M. truncatula* genome. E (expectation)-values reflect the number of hits expected by chance, so lower E-values indicate better matches. These 8 loci did not map to any genes known to be involved in the legume-rhizobia mutualism. The remaining 12 loci did not map to a specific gene in the *M. truncatula* genome (BLAST scores: 35.6 – 102; E-values: 3.00e-20 – 3.10e-1).

The results were qualitatively similar for the Ontario sample (Table 2). The BLAST scores of the top 1% of outlier SNPs (20 SNPs total) ranged from 37.4 to 111 (E-values: 5.00e-24 – 8.90e-2). Twelve of the top 1% of SNPs in the Ontario sample mapped to genes that are not known to be involved in the legume-rhizobia mutualism. The remaining eight loci did not associate with a specific gene in the *M. truncatula* annotated genome. The BLAST scores for these loci were similar to the twelve loci that did map to specific *M. truncatula* genes (score: 35.6 – 95.1; E-value: 3.00e-20 – 3.10e-1).

There were only three outlier loci that appeared in the top 1% of SNPs in both the range-wide sample and the Ontario sample (Table 3). These loci mapped to chromosomes 1, 5, and 7 in the *M. truncatula* genome, but did not map to a specific gene. No genes found within 5 or 10kb of the *M. truncatula* orthologs of these three outliers are known to be involved in the legume-rhizobia symbiosis (assuming synteny between *M. truncatula* and *M. lupulina*). The *M. truncatula* ortholog of the outlier on chromosome 5 had two genes within 5 kb, a phosphate putative gene and a Ty3/Gypsy polyprotein/retrotransposon. The ortholog of the outlier on chromosome 1 had no genes within a 5 kb window, and the ortholog of the outlier on chromosome 7 had two genes within 5 kb, a DUF247 domain protein and a Gypsy-likepolyprotein/retrotransposon putative gene. When we increased our window size to 10 kb we found more genes, but none related to infection with rhizobia. For example, the *M. truncatula* ortholog of the outlier on chromosome 5 was close to a DUF679 domain membrane protein and an alpha/beta fold hydrolase putative gene. The ortholog of the outlier on chromosome 1 had a reverse transcriptase zinc binding protein and a homeobox knotted-like protein in its 10 kb window. The ortholog of the outlier on chromosome 7 had a phosphoenolpyruvate carboxylase within its 10 kb window, along with several putative proteins.

Finally, we calculated the distance in base pairs between the *M. truncatula* orthologs of the three outlier loci found in both the range-wide and Ontario analyses and several genes involved in *Medicago*-rhizobia association. None of the symbiosis genes that we considered were close to the orthologs of any of these three outliers. Most of the symbiosis genes are located on chromosome 5, but none were close to the ortholog of the outlier locus on chromosome 5 (Table 4). The ortholog of the outlier locus on chromosome 1 was approximately 35,822 kb away from the only symbiosis gene we considered that is located on chromosome 1 (LIN). The remaining two symbiosis genes—DMI1 and DMI2—are located on chromosomes 2 and 8 (Ané *et al.* 2002), neither of which contained any outlier loci in our analysis.

**Supplemental Tables and Figures**

Table S1. Locations of *M. lupulina* and *Ensifer* populations used in population genetic analysis.

| Population ID | Province/State | Country | Latitude | Longitude |
| --- | --- | --- | --- | --- |
| AV | Pennsylvania | United States | 40.200904 | -76.763663 |
| BR | Ontario | Canada | 43.749940 | -79.639721 |
| COB | Ontario | Canada | 44.103365 | -78.156738 |
| DE | Delaware | United States | 38.686381 | -75.07443 |
| HO | Ontario | Canada | 44.219923 | -81.049833 |
| KA | Ontario | Canada | 44.555739 | -78.838577 |
| MAP | Ontario | Canada | 43.836307 | -80.636623 |
| PA1 | Pennsylvania | United States | 39.930768 | -75.584929 |
| PAR | New York | United States | 40.659773 | -76.918652 |
| PT | Pennsylvania | United States | 40.07175 | -75.435691 |
| SEG | Ontario | Canada | 45.225519 | -79.682214 |
| SIN | Pennsylvania | United States | 41.457507 | -77.135632 |
| UBR | Delaware | United States | 39.626937 | -75.675749 |
| WA | Ontario | Canada | 43.596353 | -80.625336 |
|  |  |  |  |  |

Table S2. Summary statistics of Bayenv2 and BLAST results for the top 1% of SNPs in the X^T^X outlier analysis.

| **Ontario sample** | |  |  |  |  |
| --- | --- | --- | --- | --- | --- |
| **X^T^X** | **SNP identity** | **BLAST** | **Query cover** | **E value** | **Gene** |
| 4.20 | 585117 | 37.4 | 56 | 0.089 | transmembrane protein |
| 3.83 | 1192907 | 93.3 | 100 | 1.00E-18 | indole-3-glycerol phosphate lyase IGL1 |
| 3.82 | 187811 | 93.3 | 98 | 1.00E-18 | toprim domain protein |
| 3.75 | 1110167 | 107 | 100 | 6.00E-23 | TLD-domain nuclear protein |
| 3.73 | 1610082 | 71.6 | 81 | 4.00E-12 | no result |
| 3.65 | 229813 | 35.6 | 60 | 3.10E-01 | no result |
| 3.59 | 1959186 | 107 | 100 | 6.00E-23 | TPR repeat protein |
| 3.56 | 129152 | 41 | 59 | 7.00E-03 | no result |
| 3.55 | 884266 | 89.7 | 100 | 2.00E-17 | no result |
| 3.44 | 616912 | 95.1 | 98 | 4.00E-19 | no result |
| 3.43 | 97240 | 111 | 100 | 5.00E-24 | transportin-1 protein |
| 3.39 | 666854 | 87.8 | 100 | 6.00E-17 | no result |
| 3.39 | 713735 | 96.9 | 100 | 1.00E-19 | indole-3-glycerol phosphate lyase IGL1 |
| 3.33 | 1294820 | 69.8 | 95 | 2.00E-11 | copia-like polyprotein/retrotransposon |
| 3.33 | 686219 | 69.8 | 100 | 2.00E-11 | no result |
| 3.33 | 1463455 | 91.5 | 100 | 5.00E-18 | single-stranded nucleic acid-binding protein R3H |
| 3.29 | 671122 | 37.4 | 85 | 8.90E-02 | cysteinyl-tRNA synthetase |
| 3.26 | 1071597 | 98.7 | 100 | 3.00E-20 | no result |
| 3.25 | 109965 | no result | no result | no result | no result |
| 3.25 | 734196 | 78.8 | 100 | 3.00E-14 | no result |
| **Range-wide sample** | |  |  |  |  |
| 3.25 | 825707 | 96.9 | 100 | 1.00E-19 | FAD/NAD(P)-binding oxidoreductase family protein |
| 3.06 | 1131532 | 89.7 | 100 | 2.00E-17 | novel plant SNARE-like protein |
| 3.02 | 1482582 | 35.6 | 39 | 0.31 | ASCH domain protein |
| 2.99 | 405789 | 89.7 | 93 | 2.00E-17 | CAAX amino terminal protease family protein |
| 2.99 | 511074 | 48.2 | 96 | 5.00E-05 | no result |
| 2.97 | 175058 | 55.4 | 96 | 3.00E-07 | no result |
| 2.95 | 485278 | 91.5 | 93 | 5.00E-18 | no result |
| 2.94 | 254373 | 59 | 100 | 3.00E-08 | CRS1/YhbY (CRM) domain protein |
| 2.92 | 1090953 | 55.4 | 95 | 3.00E-07 | galactose oxidase |
| 2.90 | 921907 | 93.3 | 100 | 1.00E-18 | DUF223 domain protein |
| 2.86 | 1071597 | 98.7 | 100 | 3.00E-20 | no result |
| 2.82 | 870953 | 35.6 | 51 | 3.10E-01 |  |
| 2.81 | 1590352 | 102 | 92 | 3.00E-21 | no result |
| 2.75 | 686219 | 69.8 | 100 | 2.00E-11 | no result |
| 2.73 | 774291 | 69.8 | 95 | 2.00E-11 | no result |
| 2.71 | 313573 | 69.8 | 98 | 2.00E-11 | no result |
| 2.68 | 1342844 | 60.8 | 90 | 8.00E-09 |  |
| 2.67 | 1217147 | 89.7 | 100 | 2.00E-17 | novel plant SNARE-like protein |
| 2.64 | 616912 | 84.2 | 100 | 7.00E-16 | no result |
| 2.64 | 1057909 | 66.2 | 70 | 2.00E-10 | no result |

Table S3. Outlier loci found in the top 1% of Bayenv2 results in both the *M. lupulina* range-wide and Ontario samples.

| **X^T^X** | **SNP identity** | **BLAST** | **Query cover** | **E value** | **Gene name** |
| --- | --- | --- | --- | --- | --- |
| 3.26 | 1071597 | 98.7 | 100 | 3.00E-20 | no result |
| 3.44 | 616912 | 95.1 | 98 | 4.00E-19 | no result |
| 3.33 | 686219 | 69.8 | 100 | 2.00E-11 | no result |

Table S4. Base pair distances between the *M. truncatula* ortholog of the outlier locus on chromosome 5 and well-characterized nodulation and rhizobial infection genes in *M. truncatula*.

| **Gene name** | **Distance (kb)** |
| --- | --- |
| NFP (Nod-factor receptor 5) | 12397 |
| NSP (Nodulation receptor kinase-like protein) | 6319 |
| LYK3 (LysM receptor kinase K1B) | 18 |
| NIN (Nodule inception protein) | 24 |
| IPD3 (Cyclops protein putative) | 8469 |


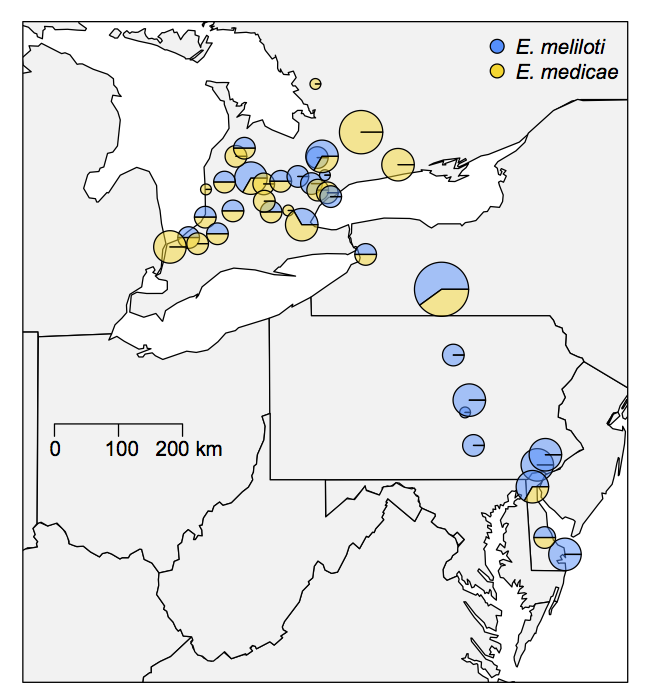


Figure S1. Locations and bacterial species present in all 39 sampled *M. lupulina* populations. The size of each circle corresponds to the number of sampled plant individuals for which their bacterial partner was identified to species. The colors correspond to the fraction of plants partnered with *E. meliloti* (blue) and *E. medicae* (yellow). The map is modified from Figure 1 in Harrison (2015).
